# Gravitational-wave constraints on the pair-instability mass gap and nuclear burning in massive stars

**DOI:** 10.1038/s41550-026-02847-0

**Published:** 2026-05-07

**Authors:** Fabio Antonini, Isobel M. Romero-Shaw, Thomas Callister, Fani Dosopoulou, Debatri Chattopadhyay, Yonadav Barry Ginat, Mark Gieles, Michela Mapelli

**Affiliations:** 1 Gravity Exploration Institute, School of Physics and Astronomy, Cardiff University, Cardiff, UK; 2 H. H. Wills Physics Laboratory, University of Bristol, Bristol, UK; 3 Kavli Institute for Cosmological Physics, The University of Chicago, Chicago, IL USA; 4 Center for Interdisciplinary Exploration and Research in Astrophysics (CIERA) and Department of Physics & Astronomy, Northwestern University, Evanston, IL USA; 5 Rudolf Peierls Centre for Theoretical Physics, University of Oxford, Oxford, UK; 6 New College, Holywell Street, Oxford, UK; 7 ICREA, Barcelona, Spain; 8 Institut de Ciéncies del Cosmos (ICCUB), Universitat de Barcelona (IEEC-UB), Barcelona, Spain; 9 Institut für Theoretische Astrophysik, Zentrum für Astronomie (ZAH), Universität Heidelberg, Heidelberg, Germany; 10 Physics and Astronomy Department Galileo Galilei, University of Padova, Padova, Italy

**Keywords:** Stellar evolution, Galaxies and clusters, General relativity and gravity

## Abstract

Pair instability should prevent the direct formation of black holes above about 50 *M*_⊙_, creating a ‘pair-instability’ mass gap. Yet gravitational-wave observations have detected black holes in this mass range. These systems can be explained with uncertainties in massive-star evolution, or hierarchical mergers in stellar clusters, which are expected to produce large spins with isotropic orientations. Here we present evidence for the pair-instability mass gap in the LIGO–Virgo–KAGRA fourth transient catalogue, with a lower edge at $44.{3}_{-3.5}^{+5.9}\,{M}_{\odot }$. We also obtain a measurement of the ^12^C(α, γ)^16^O reaction rate, yielding an *S*-factor of $26{8}_{-116}^{+195}\,{\rm{keV\; b}}$, a parameter critical for modelling helium burning and stellar evolution. The data reveal two populations: a low-spin group with no black holes above the gap, and a high-spin, isotropic group that extends across the full mass range and occupies the gap, consistent with hierarchical mergers. These findings are consistent with pair instability playing a role in shaping the black hole mass spectrum, point to a connection between gravitational-wave astronomy and nuclear astrophysics, and highlight dense stellar clusters as key environments in the growth of black holes.

## Abstract (web summary)

Gravitational-wave data reveal evidence for the pair-instability mass gap and a population of high-mass black hole mergers formed in star clusters. The measurement also constrains the nuclear reaction that regulates carbon–oxygen production in massive stars.

## Main

Gravitational-wave observations of binary black holes have opened a new window onto massive-star evolution^1–5^, but population inferences remain hampered by uncertainties in binary physics and initial conditions (see, for example, refs. ^6–9^). A central issue is whether (pulsational) pair-instability supernovae (PISNs) carve out a gap in the black hole birth mass distribution (see, for example, refs. ^10–12^); theory predicts pulsations for He cores of ~40–65 *M*_⊙_ and full disruption above ~65 *M*_⊙_, suppressing black hole formation in the ~40–130-*M*_⊙_ range^13–18^. Gravitational-wave observations have so far revealed no sharp deficit of black holes in this mass range (the so-called PISN mass gap)^2,3,19–23^, motivating scenarios that populate the gap (see, for example, refs. ^11,15,24,25^), or raising the possibility that the gap may not exist at all (see, for example, ref. ^26^).

Dynamical environments (for example, globular and nuclear clusters or active galactic nucleus disks) can produce merger remnants that merge again, yielding higher spins^27–30^ with isotropic orientations^31–33^, and populating the PISN mass gap. For binaries in which the primary component was produced by a previous merger, the effective combination of the two component spins projected parallel to the orbital angular momentum^34,35^, *χ*_eff_ (the best measured spin parameter from data), is expected to be broad and symmetric around zero. Reference ^36^ showed that this distribution is largely independent of model assumptions and uncertainties, and derived an approximately uniform form with ∣*χ*_eff_∣ ≲ 0.5. This bound follows from the fact that merger remnants are expected to have a nearly universal dimensionless spin magnitude, *a*_rem_ ≃ 0.7, as predicted by general relativity^27^. Assuming a negligibly spinning companion, *χ*_eff_ satisfies $| {\chi }_{{\rm{eff}}}| \lesssim \frac{{m}_{{\rm{rem}}}{a}_{{\rm{rem}}}}{{m}_{{\rm{rem}}}+{m}_{2}},$ where *m*_rem_ and *m*_2_ are the remnant and secondary black hole masses, respectively. For *m*_2_ ≃ 0.5 *m*_rem_, as expected in dynamical formation^37^, this yields ∣*χ*_eff_∣ ≲ 0.47. The presence and location of the PISN lower mass limit $\widetilde{m}$ can therefore be inferred from the primary mass *m*_1_ at which the *χ*_eff_ distribution transitions to this broad, symmetric form.

We perform hierarchical Gaussian-process population inference on the fourth LIGO–Virgo–KAGRA gravitational-wave transient catalogue (GWTC-4)^38,39^ to map black hole spin as a function of primary mass. With the source catalogue now more than twice as large, we obtain tight constraints and are able to probe new features of the population. We fit the *χ*_eff_ distribution to a mixture model comprising a Gaussian distribution, representing the bulk of the population at ${m}_{1}\lesssim \widetilde{m}$, and a higher-mass spin distribution described via a non-parametric Gaussian-process prior.

**Fig. 1:**
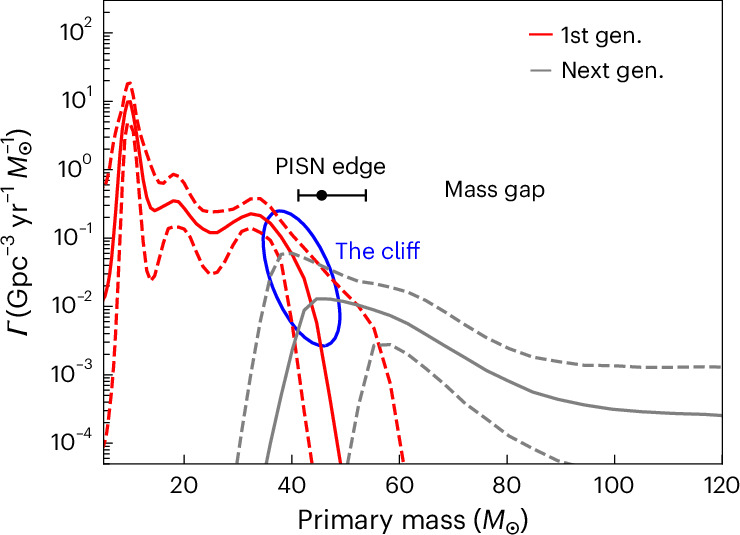
The primary black hole mass spectrum. Merger rate as a function of primary black hole mass in binaries below (red) and above (grey) the truncation mass $\widetilde{m}$ separating low- and high-spin populations, calculated at a reference redshift *z* = 0.2. Given the merger-rate density per unit primary mass at the reference redshift, *Γ*(*m*_1_), the primary-mass distribution for the two populations below and above $\widetilde{m}$ are reconstructed from the posterior samples as ${\varGamma }_{ < \widetilde{m}}({m}_{1})=\left[1-\eta ({m}_{1})\right]\varGamma ({m}_{1})$ and ${\varGamma }_{ > \widetilde{m}}({m}_{1})=\eta ({m}_{1})\varGamma ({m}_{1})$, respectively. Here *η*(*m*_1_) is a sigmoid mixing function that sets the relative contribution of the two population components as a function of primary mass, with $\eta (\widetilde{m})=0.9$. This model yields a total merger rate at this redshift of $33.{4}_{-8.4}^{+13.3}\,{{\rm{Gpc}}}^{-3}\,{{\rm{yr}}}^{-1}$, consistent with ref. ^40^. The inferred $\widetilde{m}$ is marked by the black symbol. Solid lines indicate the median merger rate and dashed lines the 10th–90th percentiles. A mass gap in the low-spin population, isotropic spins above $\widetilde{m}$ and a sharp drop in the merger rate at the same mass value (the cliff) are all features consistent with a PISN gap populated by hierarchical mergers in dense star clusters.

We identify a transition at $\widetilde{m}=45.{6}_{-5.6}^{+12.7}\,{M}_{\odot }$ (90% confidence), separating the two populations. The differential merger rates as a function of primary mass for the two populations are given in Fig. 1, while the inferred spin distributions are given in Supplementary Information. Below $\widetilde{m}$, the data are well described by a single narrow Gaussian *χ*_eff_ distribution, ${\log }_{10}\,\sigma =-1.1{5}_{-0.15}^{+0.13}$, with small and positive mean, $\mu =0.0{4}_{-0.02}^{+0.02}$, consistent with first-generation black holes. The merger rate of this population drops to zero above $\widetilde{m}$, implying a gap in the mass spectrum of first-generation mergers. In contrast, the population above $\widetilde{m}$ exhibits a much broader spin distribution with a median value $\langle {\chi }_{{\rm{eff}}}\rangle =0.1{1}_{-0.243}^{+0.197}$. Under the proposed model, the data are consistent with a *χ*_eff_ distribution that is symmetric about zero and with the expected distribution of second-generation mergers formed dynamically in dense stellar environments.

The precise measurement of $\widetilde{m}$ means that the mixture model is strongly favoured over models in which black holes of all masses share the same spin distribution. To quantify support for the transition-mass model, we compute the Bayes factor relative to the default model used by the LIGO–Virgo–KAGRA collaboration (see, for example, ref. ^40^), in which the full *χ*_eff_ distribution is described by a single truncated normal, $P({\chi }_{\mathrm{eff}})={\mathcal{N}}({\chi }_{\mathrm{eff}};\,\mu ,\sigma )$, with no distinct high-mass component. We sample this hierarchical model using the same event set, likelihood and inference framework as in the main analysis. We obtain a Bayes factor *B* > 10^4^ in favour of the model with a separate high-mass spin component.

The overall mass distribution shows several features. There are peaks at ~10 *M*_⊙_, 18 *M*_⊙_ and 38 *M*_⊙_, as also reported in ref. ^40^—these peaks were previously identified in ref. ^41^. There is a drop of nearly two orders of magnitude in the total merger rate at ~40 *M*_⊙_ (we name this feature ‘the cliff’ in Fig. 1). A rapid decline in the merger rate followed by a plateau (or a shallower decline) at $\widetilde{m}$, as we found, is a generic feature of cluster formation models^36,42^. The plateau starts at the onset of PISNs and it is due to the emergence of binaries with components formed from previous mergers. According to these models, the edge of the transition between the two populations can be estimated from the primary-mass distribution alone as the value of maximum curvature above 30 *M*_⊙_ in the differential merger rate, *Γ*(*m*_1_) (‘The pair-instability mass gap in star-cluster models’). We measure this transition from the data at ~42 *M*_⊙_, consistent with the independent estimate based on the spin transition. A comprehensive interpretation of the cliff may require accounting for multiple concurring physical processes, as discussed in Supplementary Information.

**Fig. 2:**
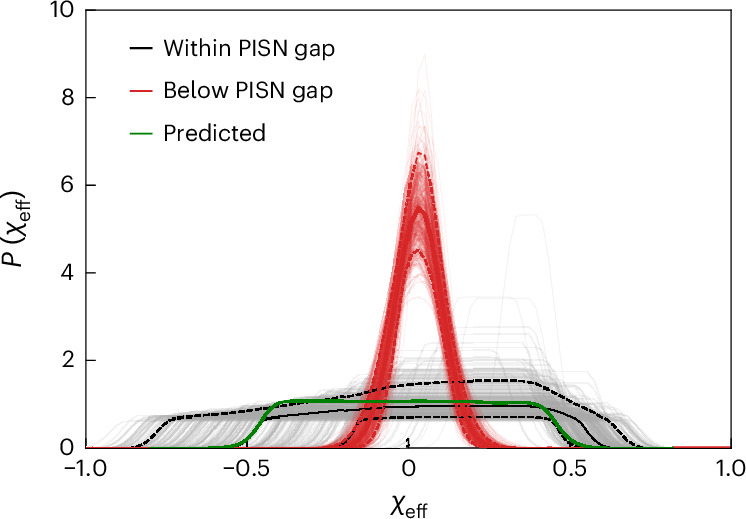
The *χ*_eff_ distributions below and within the PISN mass gap. Thick black lines show the *χ*_eff_ distribution for the high-mass population, $m > \widetilde{m}$, modelled as a uniform distribution with independent bounds, while thick red lines correspond to the *χ*_eff_ distribution of the low-mass population, $m < \widetilde{m}$. Solid lines are the medians, while dashed lines show 10% and 90% of the distributions. The distribution for *χ*_eff_ in the PISN mass gap predicted under a hierarchical formation scenario is shown in green^42^. Light colour traces correspond to a single draw from the posterior distribution, providing a visual representation of the sample support from which the confidence intervals are constructed.

The non-parametric analysis over a large dataset gives us confidence that a transition to a broader and more uniform distribution exists in the population. Motivated by this, we introduce a more informed parametric model in which the high-mass population is described by a uniform distribution with independent bounds, and use this model in what follows (unless otherwise specified). As before, the distribution below $\widetilde{m}$ is well described by a normal distribution with positive mean, $\mu =0.0{5}_{-0.02}^{+0.02}$, and small dispersion, ${\log }_{10}\sigma =-1.2{9}_{-0.17}^{+0.18}$. The distribution above $\widetilde{m}$ is a broad distribution for which we infer upper and lower bounds of ${\chi }_{{\rm{eff,max}}}=0.4{9}_{-0.12}^{+0.14}$ and ${\chi }_{{\rm{eff,min}}}=-0.4{1}_{-0.35}^{+0.40}$, respectively. The lower bound is less well constrained than the upper bound, primarily due to selection effects and parameter-estimation uncertainties. Nevertheless, we find that *χ*_eff,min_ < 0 with 98.4% credibility, providing strong evidence for misaligned spins in the population. The median value $\langle {\chi }_{{\rm{eff}}}\rangle =0.0{2}_{-0.17}^{+0.18}$ indicates that the distribution is statistically consistent with being symmetric around zero. Figure 2 shows the corresponding recovered *χ*_eff_ distributions.

**Fig. 3:**
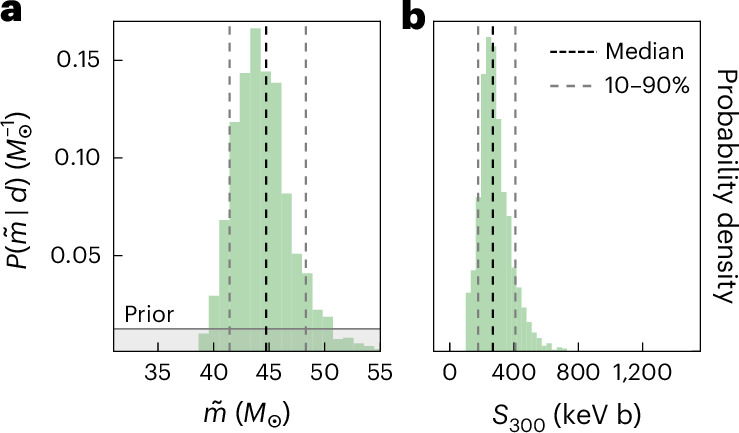
Constraints on the PISN transition mass and the ^12^C(α, γ)^16^O reaction rate. **a**, Posterior distribution of the primary-mass value separating the two black hole populations, $\widetilde{m}$. **b**, The corresponding posterior of *S*_300_. The latter is derived from $\widetilde{m}$, using this as the value of the lower edge of the PISN mass gap.

From the same parametric model we infer a characteristic mass scale of $\widetilde{m}=44.{3}_{-3.5}^{+5.9}\,{M}_{\odot }$, a value close to the lower edge of the pair-instability mass gap reported in theoretical and numerical studies^13,16–18^. The posterior distribution of $\widetilde{m}$ is shown in Fig. 3a.

Our results are consistent with a depletion of first-generation, low-spin black holes above $\widetilde{m}\simeq 45\,{M}_{\odot }$. We reported a similar transition at $4{6}_{-6}^{+7}\,{M}_{\odot }$ in GWTC-3 using 69 sources, with 11 having 90% of the *m*_1_ posterior distribution above 45 *M*_⊙_ after population reweighting^36^, and a transition to a higher-spin population was identified by others^43,44^. The consistent recovery in the new, larger catalogue containing 153 sources, with 34 above 45 *M*_⊙_, demonstrates that the feature is strongly driven by the data and it is not a statistical fluctuation. Constraints on the *χ*_eff_ distribution of the high-mass population are now tighter and more consistent with our theoretical interpretation. Using GWTC-3 we inferred ${\chi }_{{\rm{eff,max}}}=0.5{7}_{-0.19}^{+0.21}$ (uniform model), and could not decisively exclude *χ*_eff,max_ = 1 or *χ*_eff,max_ < 0 (ref. ^45^). With the expanded dataset we can instead rule out both *χ*_eff,max_ = 1 and *χ*_eff,max_ < 0 and infer *χ*_eff,max_ ≈ 0.5. This is notable because hierarchical mergers are the only astrophysical pathway that robustly predicts this upper bound^36^. The low-*χ*_eff_ tail is also better constrained. In GWTC-3, *χ*_eff,min_ and the left-hand tail were sensitive to prior choices (see, for example, Fig. 5 in ref. ^45^); with the larger sample we find −1 < *χ*_eff,min_ < 0 with substantially higher confidence, and a peak near the expected value *χ*_eff,min_ ≈ −0.5.

Together, these robust features indicate that the data are consistent with a hierarchical origin of the high-mass population: a mass gap in the low-mass/low-spin population, the onset of an isotropic and highly spinning population above $\widetilde{m}$, the sharply defined upper bound of the *χ*_eff_ distribution at ~0.5 and the steep decline in the total merger rate (the cliff) near the transition. The transition mass itself, $\widetilde{m}\simeq 45\,{M}_{\odot }$, matches stellar-evolution predictions for the onset of the pair-instability mass gap. Recent studies^46,47^ have identified a sharp decline in the merger rate—or possibly even a ‘gap’, as suggested by concurrent work^48^—for systems with secondary masses above ~45 *M*_⊙_, which is expected due to the rarity of binaries in which both components are second-generation black holes. Although PISN-gap mergers are expected mainly to involve a first-generation black hole and a merger remnant, we note that binaries where both black holes are merger remnants should also occur, as has been suggested for GW190521 (see, for example, ref. ^49^). Together, these independent lines of evidence favour hierarchical mergers as the most likely origin of the high-mass population. Such signatures are difficult to explain through isolated binary evolution, but arise naturally if the high-mass population is built from hierarchical mergers in dense stellar environments. With this level of confidence, we can now use this result to place direct constraints on massive-star evolution and the physics of the pair-instability process.

The location of the PISN boundary is ultimately set by stellar evolution physics, and in particular by the relative abundances of carbon and oxygen in the cores of very massive stars before collapse. These abundances depend on the ^12^C(α, γ)^16^O reaction rate, which governs the conversion of carbon into oxygen during helium burning^13,16–18^. A higher rate enhances oxygen production, leading to larger oxygen-rich cores and, consequently, to PISNs occurring at lower stellar masses. Conversely, a lower rate leaves behind more carbon, shifting the onset of pair instability to higher progenitor masses. Thus, measurements of the PISN mass gap from gravitational-wave observations of black hole mergers can provide an astrophysical constraint on the ^12^C(α, γ)^16^O cross-section, a quantity that remains one of the most important nuclear-physics uncertainties in massive stellar modelling^15,50^.

We assume that $\widetilde{m}$ is the lower edge of the PISN mass gap, and follow refs. ^15,51^ to translate our inferred $\widetilde{m}$ posterior into an estimate of the corresponding astrophysical *S*-factor at 300 keV, *S*_300_. The astrophysical *S*-factor rewrites a nuclear reaction cross-section by factoring out the strong Coulomb-barrier dependence, $\sigma (E)=\frac{S(E)}{E}\,{{\rm{e}}}^{-2{\rm{\pi }}\eta }$, where *E* is the centre-of-mass energy and *η* the Sommerfeld parameter. We obtain ${S}_{300}=26{8}_{-116}^{+195}\,{\rm{keV\; b}}$ (90% credibility); we plot this probability distribution in Fig. 3. This estimate is consistent, within uncertainties, with recent nuclear-physics determinations^52–54^.

Our inference of the ^12^C(α, γ)^16^O *S*-factor from gravitational-wave data provides a novel, astrophysical constraint on a parameter that has long been central to stellar evolution theory. It relies solely on the assumption that the population with mass of ≳45 *M*_⊙_ consists entirely of second- (or higher-) generation black holes. Although direct nuclear-physics experiments have yielded estimates with large uncertainties^15^, our measurement achieves substantially tighter bounds, enabled by the sensitivity of the black hole mass spectrum to the details of helium burning. This improvement has wide-ranging implications: the carbon-to-oxygen ratio set by this reaction influences the core structure of massive stars, and thus affects the predicted rate of core-collapse supernovae, the maximum masses of neutron stars and the fate of red supergiants. It also governs the composition of white dwarfs (see, for example, ref. ^55^), with consequences for type Ia supernova explosions (see, for example, ref. ^56^), and shapes the nucleosynthetic yields that feed into galactic chemical evolution (see, for example, ref. ^57^). More broadly, the balance between carbon- and oxygen-rich material determines the conditions for planet formation and the likelihood of forming C-rich versus O-rich planetary systems (see, for example, ref. ^58^). Gravitational-wave astronomy therefore not only constrains the physics of compact objects, but also offers a new window into the nuclear processes that regulate stellar evolution and the chemical enrichment of the Universe.

**Fig. 4:**
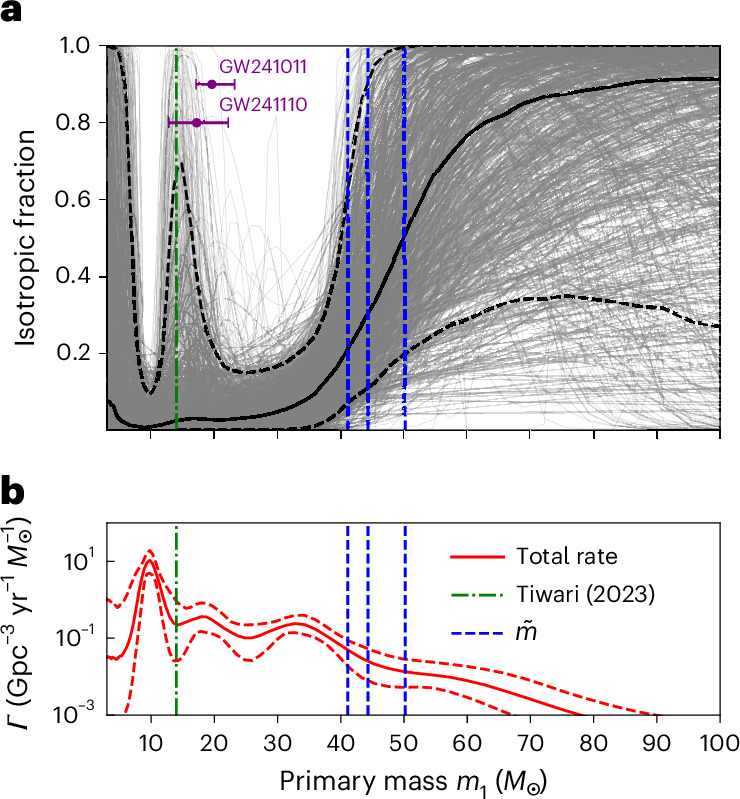
Mass-dependent mixture fraction between the high- and low-spin populations. **a**, The mixture fraction between the two black hole populations, modelled non-parametrically as a function of mass. The median and the 10% and 90% percentiles are shown. Light black lines are individual traces. **b**, The total differential merger rate as a function of primary black hole mass for reference. A transition above ~50 *M*_⊙_ is clearly recovered by this analysis. We also find a possible indication of an isotropic population at a primary mass of ~14 *M*_⊙_, although it is not statistically required by the data. This aligns with the mass dip in ref. ^59^ (green) and with GW241011 and GW241110, two O4b events interpreted as hierarchical mergers^82^ (pink). Together, these support our interpretation of a low-mass ‘valley’, which is populated by hierarchical mergers. Below ~10 *M*_⊙_, the posterior broadens, reverting to the prior due to the absence of sources there.

We now use a non-parametric approach to search for additional isotropically spinning components in the data and to further test our result of a transition in spin properties at *m*_1_ ≃ 45 *M*_⊙_. We model the mixture fraction between the low-spin and the high-spin and isotropic populations as a non-parametric function of the primary mass. The posterior distribution of the mixture fraction is shown in Fig. 4, indicating that the fraction of isotropically spinning binaries is consistent with a sharp increase above ≳45 *M*_⊙_.

A new feature appears at *m*_1_ ≃ 14 *M*_⊙_, where the 90% bound of the mixing fraction rises to ~0.6. This coincides with a possible dip in the merger rate—previously identified in refs. ^41,59^. We interpret this as marginal evidence for an additional lower-mass gap in first-generation black holes that is populated by black holes formed from a previous merger^60^. While consistent with current data, this feature is not statistically required. Applying the same model to GWTC-3^4^ yields an upper bound of ~0.16, showing that this feature only becomes discernible with the larger GWTC-4 catalogue.

The data indicate that nearly all primary black holes above 45 *M*_⊙_ involved in binary mergers possess high, isotropic spins. Explaining this within stellar evolution would require a mechanism that produces mass-dependent black hole spins at the end of massive-star lifetimes while also overcoming the pair-instability gap. The latter might be achieved through reduced stellar winds at low metallicity combined with the collapse of the residual hydrogen-rich envelope during a failed supernova^26,61^, but no explanation currently exists for the former. Another possibility is that stellar evolution could generate rapidly rotating black holes above ~45 *M*_⊙_ through fallback of angular-momentum-rich envelopes.

Our preferred explanation is that primary black holes above 45 *M*_⊙_ are the products of repeated mergers in globular clusters^42^. The inferred merger rate above this mass therefore provides a strong constraint on the initial cluster density: if all mergers above 45 *M*_⊙_ have this origin, the models of ref. ^42^ imply formation densities of ≳10^4^ *M*_⊙_ pc^−3^. Repeated mergers may also occur in active galactic nucleus disks^62^ or nuclear star clusters^32^. Because these environments have higher escape velocities than do globular clusters, the detailed shape of the *m*_1_ distribution could help determine their relative contributions. After our work appeared on arXiv, ref. ^63^ likewise found a zero-symmetric *χ*_eff_ distribution above 45 *M*_⊙_. They showed that the two populations can also be distinguished by their mass-ratio distributions, with the higher-mass population favouring more asymmetric binaries, consistent with hierarchical formation in clusters. They noted, however, that the mass-ratio distribution of the high-mass population was not reproduced by their particular globular-cluster models. This could reflect either uncertainties in the cluster models and in the limited parameter space explored, or additional channels contributing within the PISN gap.

As the catalogue of detected binary black holes continues to expand with future observing runs, constraints on the pair-instability mass gap will sharpen, enabling increasingly stringent bounds on the ^12^C(α, γ)^16^O cross section. In the coming years, gravitational-wave population inference will thus not only elucidate the astrophysical environments where black holes form and merge, but also offer a new avenue to constrain fundamental nuclear reaction rates that underpin the evolution and fate of massive stars. At the same time, the identification of a population formed in dense star clusters offers a powerful opportunity to probe their initial conditions and evolutionary pathways across cosmic time.

## Methods

### Population models

We consider the subset of binary black hole mergers in GWTC-4 with false alarm rates below 1 yr^−1^, consistent with ref. ^40^. The data we used are public open data from the LIGO–Virgo–KAGRA collaboration^39,64^.

For events originally published in GWTC-1^1^, we use the ‘Overall_posterior’ parameter-estimation samples. For events originally published in GWTC-2^5^ and ref. ^65^, we adopt the ‘PrecessingSpinIMRHM’ samples, for events in GWTC-3^4^ we use the ‘C01:Mixed’ samples available from ref. ^66^ and for events in GWTC-4 we use the NRSur7dq4 samples if available^67^, or the ‘Mixed’ samples otherwise. We exclude the events which include at least one component with mass of <3 *M*_⊙_ and are therefore likely to involve a neutron star^20,39,40^. This results in 153 events. The detections in GWTC-4 were enabled by a variety of detector improvements^68–73^. Selection effects are accounted for using the set of successfully recovered binary black hole injections made publicly available by the LIGO–Virgo–KAGRA collaboration, covering their first four observing runs^20,40,74^. Thus, our analysis accounts for selection effects and measurement uncertainties through the hierarchical Bayesian inference framework, which models the population distribution while marginalizing over individual-event posteriors.

We assume that the merger-rate density factorizes as

1 $$R({m}_{1},{m}_{2},{\chi }_{\mathrm{eff}};z)={R}_{\mathrm{ref}}\,\frac{f({m}_{1})}{f(20\,{M}_{\odot })}{\left(\frac{1+z}{1.2}\right)}^{\kappa }P({m}_{2}| {m}_{1})\,P({\chi }_{\mathrm{eff}}| {m}_{1}),$$

where *R*_ref_ is the rate per unit mass at *m*_1_ = 20 *M*_⊙_ and *z* = 0.2. Our main focus is the conditional spin distribution *P*(*χ*_eff_∣*m*_1_), for which we consider a flexible non-parametric model.

In our analysis we simultaneously infer the distributions of binary black hole primary masses *m*_1_, mass ratios *q* and redshifts *z*. We model the conditional distribution of *m*_2_ as (see, for example, ref. ^75^)

2 $$P({m}_{2}| {m}_{1})\propto {m}_{2}^{{\beta }_{q}},\,2{M}_{\odot }\le {m}_{2}\le {m}_{1}.$$

Meanwhile, we assume that the volumetric merger rate evolves as a power law in (1 + *z*) (refs. ^76,77^), such that probability distribution of merger redshifts is

3 $$P(z)\propto \frac{1}{1+z}\frac{{\rm{d}}{V}_{{\rm{c}}}}{{\rm{d}}z}{(1+z)}^{\kappa },$$

where *V*_c_ is the comoving volume. In all models, the primary-mass spectrum is modelled non-parametrically with a Gaussian process (GP)^78^: $$f({m}_{1})=\exp [\varPhi (\mathrm{ln}\,{m}_{1})],$$$$\varPhi (x)\approx \mathrm{GP}(0,\,k(x,{x}^{{\prime} };{a}_{m},{\ell }_{m})),$$ with a squared-exponential kernel. Here, *a*_*m*_ is the amplitude of the GP (controlling vertical variation) and *ℓ*_*m*_ is the length scale (controlling smoothness), which are treated as free hyperparameters. The GP is evaluated on a uniform grid in log *m*_1_ between 2 and 200 *M*_⊙_, and interpolated to event samples and injections.

We model the *χ*_eff_ distribution as a mixture of two components: a truncated Gaussian between [−1, 1], describing the bulk of the population at ${m}_{1}\lesssim \widetilde{m}$, and a flexible non-parametric distribution at ${m}_{1}\gtrsim \widetilde{m}$. The parameter $\widetilde{m}$ marks the transition between the two regimes:

4 $$\begin{array}{rcl}P({\chi }_{\mathrm{eff}}| {m}_{1})&=&\left[1-\eta ({m}_{1})\right]\,{\mathcal{N}}({\chi }_{\mathrm{eff}};\mu ,\sigma )+\eta ({m}_{1})\,\exp \left[\varTheta ({\chi }_{\mathrm{eff}})\right]/ \\ && \int_{-1}^{1}\exp \left[\varTheta ({\chi }_{\mathrm{eff}})\right]\mathrm{d}\chi_{\rm eff},\end{array}$$

where

5 $$\eta ({m}_{1})={\left[1+\frac{1}{9}\exp \left(-\frac{({m}_{1}-\widetilde{m})}{{M}_{\odot }}\right)\right]}^{-1}.$$

This choice ensures that $\eta (\widetilde{m})=0.9$, that is, at ${m}_{1}=\widetilde{m}$ the Gaussian component contributes 10% of the total, while the non-parametric component dominates above the transition. The function *Θ*(*χ*_eff_) is generated from a GP, $\varTheta ({\chi }_{\mathrm{eff}})\approx \mathrm{GP}(0,\,k({\chi }_{\mathrm{eff}},{\chi }^{{\prime} };\,{a}_{\chi },{\ell }_{\chi }))$, with zero mean and a squared-exponential covariance kernel. We evaluate these GPs on a regular grid of *N*_bin_ = 100 points in *χ*_eff_ within the range from −1 to +1, following ref. ^45^. We verified that our non-parametric model is sufficiently flexible to recover a narrow *χ*_eff_ distribution if that is what the data prefer. In particular, we model the full population with a GP prior, that is, we repeat the inference using a model in which *P*(*χ*_eff_) = exp[*Θ*(*χ*_eff_)]. The GP contracts to an approximately Gaussian, narrow distribution. This demonstrates that the broader, mass-dependent behaviour we infer is data driven rather than imposed by the model.

Similarly, in the main text we consider a model where the mixing fraction between the two populations, *ζ*, is a non-parametric function of *m*_1_ (ref. ^45^). Here *ζ*(*m*_1_) denotes the fraction of binaries with isotropic spin orientations, such that $$P({\chi }_{\mathrm{eff}}\,|\,{m}_{1})=(1-\zeta ({m}_{1}))\,N({\chi }_{\mathrm{eff}};\mu ,\sigma )$$$$+\zeta ({m}_{1})\,U({\chi }_{\mathrm{eff}};w)$$, and $${U}({\chi }_{{\rm{eff}}};w)$$ is a uniform distribution over ∣*χ*_eff_∣ < *w*, with *w* the width of the distribution that is also recovered from the data. In this model we set

6 $$\zeta ({m}_{1})=S[\varPsi (\mathrm{ln}\,{m}_{1})],\,\,\,\,\,\,\,\,\,\,\,\,\,\,\,\,\varPsi (x)\approx GP(0,\,k(x,{x}^{{\prime} };{a}_{\zeta },{\ell }_{\zeta })),$$

where the sigmoid function

7 $$S(x)=\frac{1}{1+{{\rm{e}}}^{-x}}$$

is applied to ensure 0 ≤ *ζ*(*m*_1_) ≤ 1.

In our analysis we adopt an additional effective spin model that transitions from a Gaussian to a uniform distribution below and above $\widetilde{m}$, respectively. We treat the bounds of the uniform component, *χ*_eff,min_ and *χ*_eff,max_, as free parameters inferred from the data:

8 $$P({\chi }_{{\rm{eff}}}\,|\,{m}_{1})=[1-\eta ({m}_{1})]\,{N}({\chi }_{{\rm{eff}}};\mu ,\sigma )+\eta ({m}_{1})\,{U}({\chi }_{{\rm{eff}}};\,{\chi }_{{\rm{eff}},\min },{\chi }_{{\rm{eff}},\max }).$$

We place uniform priors on these bounds: ${\chi }_{{\rm{eff}},\max }\approx {U}(0.05,1)$ and ${\chi }_{{\rm{eff}},\min }\approx {U}(-1,{\chi }_{{\rm{eff}},\max })$. We plot the joint distribution of the primary black hole mass *m*_1_ and the effective inspiral spin parameter *χ*_eff_ under this model in Supplementary Fig. 1.

The priors of the hyperparameters of our models are given in Supplementary Table 1.

### The pair-instability mass gap in star-cluster models

In the presence of a PISN mass gap, a feature might be expected to appear in the primary-mass distribution at its lower boundary due to a drop in the total merger rate (see, for example, ref. ^79^). Using cluster models, we show here that this feature takes the form of a transition in the primary-mass distribution, where the merger rate shifts from a steep decline to a flat or even rising trend. The mass at which this transition occurs provides an approximate estimate of the lower edge of the gap. We show that a similar transition is present in the observational data, and that the inferred mass scale is consistent with the value obtained from the spin transition.

For the gravitational-wave rate ${\mathcal{R}}({m}_{1})$, we compute the second derivative of the logarithm as

9 $$k({m}_{1})\equiv \frac{{{\rm{d}}}^{2}\,\log \,{R}}{{\rm{d}}{(\log \,{m}_{1})}^{2}}\,,$$

and the curvature

10 $$\kappa ({m}_{1})\equiv \frac{{{\rm{d}}}^{2}{\mathcal{R}}}{{\rm{d}}{m}_{1}^{2}}{\left[\max {{\mathcal{R}}}^{2}+{\left(\frac{{\rm{d}}{\mathcal{R}}}{{\rm{d}}{m}_{1}}\right)}^{2}\right]}^{-3/2}.$$

While *k* is dimensionless, *κ* corresponds to the standard definition of the curvature of a curve, and has dimensions of [Mass]^−2^. If there is a mass m_*_ where the rate ceases to be dominated by one population, for example first-generation mergers in clusters, and starts to become dominated by another, for example second-generation mergers in clusters, and if each population corresponds to a rate that is approximately a power law, it is natural to expect the derivative of ${\mathcal{R}}$ to change abruptly at the transition between the two populations. Thus, the maxima of *k* or *κ* would be around m_*_.

To investigate this, we consider the suite of cluster-population-synthesis simulations of ref. ^42^ that were obtained from the cluster population code cBHBd, and which explored a range of model parameters. These models have been shown to provide a good match to the observed primary-mass distribution and rate inferred from the GW data above ~30 *M*_⊙_. For each simulation we computed *k* and *κ*, varying the initial cluster half-mass density, the black hole spin distribution and the inclusion or omission of tidal interaction with an external galactic field. The ‘zero-spin’ models were run using the cBHBd settings used in ref. ^42^; the ‘no-tides’ models disabled tidal evolution entirely, and in the ‘beta-function spin’ models the initial spins were drawn from a Beta(2, 0.5) distribution. For each of these three models we explored a grid of 25 initial cluster half-mass densities, ρ_h*0*_, sampled from 10^4^ to 10^6.5^ *M*_⊙_ pc^−3^. For each density, we ran 27,216 cluster models—spanning cluster masses from 10^2^ to 10^7^ *M*_⊙_, 25 metallicities between 1.26 × 10^−4^ and 0.025, and a range of formation redshifts. To mitigate statistical fluctuations, we ran each cluster model with ten distinct initial random seeds and averaged the results. Finally, the primary-mass distribution is reconstructed by forward-modelling the full cluster population as in ref. ^42^: we sample dynamical cluster models within a cosmological framework that includes cluster formation histories, metallicity evolution and the cluster mass function, and use these to generate predictions for the detected astrophysical population. We smoothed the resulting mass distribution using a Gaussian kernel with width *σ*_*m*_ = 2*M*_⊙_, though the results are insensitive to the precise choice of smoothing.

For the cBHBd simulations, the maximum first-generation primary mass is m_*_ = 50 *M*_⊙_. Supplementary Fig. 2 displays a histogram of the value of *m*_1_ where these measures of the curvature attain their maxima. The maxima are obtained very close to the cutoff mass m_*_ = 50 *M*_⊙_, with remarkably small variance, irrespective of the model used; this leads us to conclude that this is a robust feature of the cluster channel. For *k*(*m*_1_), we find that the maximum overestimates the value of m_*_ = 50 *M*_⊙_ expected if *ρ*_*h*0_ > 3 × 10^5^ *M*_⊙_ pc^−3^; this is because low-mass second-generation primaries, whose progenitors were below the first-generation peak, become sufficiently prominent at high cluster densities. This is only a problem for *k*, and not for *κ*, because the former is a logarithmic measure, rendering it less sensitive to variations in ${\mathcal{R}}({m}_{1})$ than is *κ*(*m*_1_).

We compute *k* and *κ* from our non-parametric reconstruction of *Γ*(*m*_1_) inferred from the GW data. If the observations are consistent with a PISN mass gap that is populated by hierarchical mergers, both quantities might be expected to exhibit a maximum in the vicinity of $\widetilde{m}$. For each posterior trace we evaluate *k* and *κ* over the range *m*_1_ > 30 *M*_⊙_ and reconstruct the posterior distribution of the peak location, from which we extract the median and credible intervals. We obtain ${m}_{* }(k)=42.{3}_{-4.3}^{+10.1}\,{M}_{\odot }$ and ${m}_{* }(\kappa )=40.{1}_{-2.2}^{+4.1}\,{M}_{\odot }$. These values are consistent with the transitional mass $\widetilde{m}$ inferred from the *χ*_eff_ analysis, providing an independent test of the hypothesis that the observed transition is driven by the emergence of a distinct population above this mass.

## Supplementary information

Supplementary Information Supplementary Figs. 1–4, Table 1 and Discussion.

Peer Review File

## Data Availability

The data underlying this article can be downloaded at ref. ^80^.

## Code availability

The codes underlying this article can be found at https://github.com/antoninifabio/spin-study-in-O4a (ref. ^81^).
