# Supplementary material for: Gravitational-wave constraints on the pair-instability mass gap and nuclear burning in massive stars
Source: Nat Astron. 2026 May 7;10(7):1049–56. doi: 10.1038/s41550-026-02847-0 (PMC13379312; doi:10.1038/s41550-026-02847-0)
Supplement: Supplementary file 1 — Supplementary Figs. 1–4, Table 1 and Discussion. [file 41550_2026_2847_MOESM1_ESM.pdf]

nature astronomy

Supplementary information https://doi.org/10.1038/s41550-026-02847-0

# Gravitational-wave constraints on the pair-instability mass gap and nuclear burning in massive stars

In the format provided by the authors and unedited

# Supplementary Information

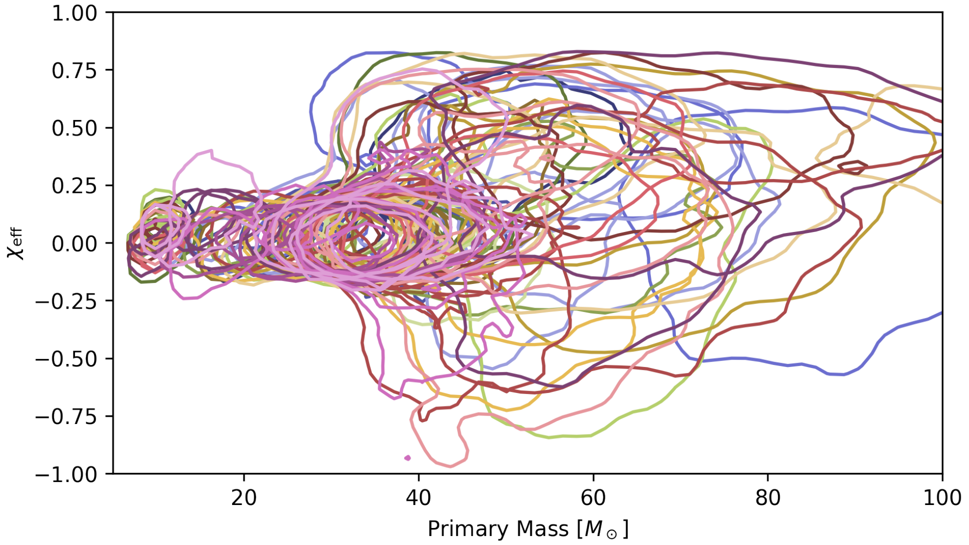


**Supplementary Figure 1**: Contours of the reweighted joint distribution of the primary black hole mass $m_1$ and the effective inspiral spin parameter $\chi_{\rm eff}$. For each event, we compute a two-dimensional kernel density estimate over $(m_1, \chi_{\rm eff})$ from the reweighted posterior samples, and plot the 95% credible region. Different colors correspond to different events. We fit the population to a model where the $\chi_{\rm eff}$ distribution is represented by a truncated Gaussian and a uniform distribution separated by mass.

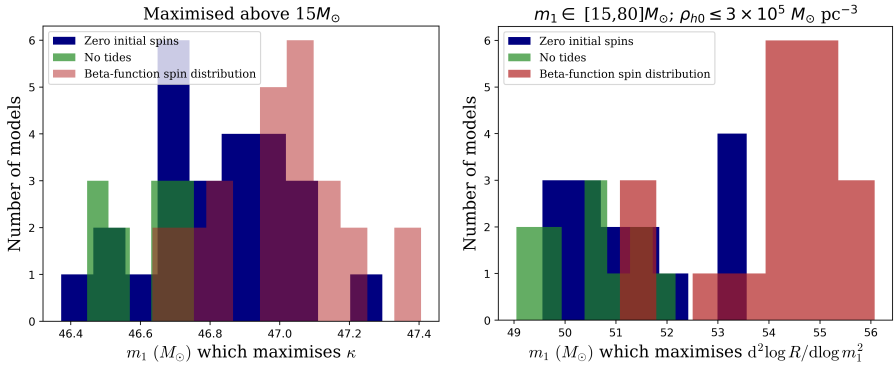


**Supplementary Figure 2**: *Left*: Histogram of the value of $m_1$ that maximizes $\kappa$ under various cluster population assumptions. *Right*: Histogram of the value of $m_1$ that maximises $k$ for the same set of models, restricted to an initial half-mass density $\rho_{h0} \leq 3 \times 10^5\, M_\odot\, \mathrm{pc}^{-3}$. Each point in the histogram corresponds to a realization of an evolved cluster population for one of the 25 assumed initial half-mass densities. For these models, the first-generation primary cutoff mass is fixed to $m_* = 50\, M_\odot$.

| Parameter | Prior | Defined in |
|---|---|---|
| $a_\chi$ | $\mathcal{HN}(3)$ | equation 4 |
| $\ln \ell_\chi$ | $\mathcal{N}(-0.5, 1)$ | equation 4 |
| $a_m$ | $\mathcal{HN}(3)$ | Mass model |
| $\ln \ell_m$ | $\mathcal{N}(0, 1)$ | Mass model |
| $\tilde{m}$ | $\mathcal{U}(20, 100)$ | equations 5 |
| $\mu$ | $\mathcal{U}(-1, 1)$ | equations 4 and 8 |
| $\sigma$ | $\mathcal{LU}(-1.5, 0)$ | equations 4 and 8 |
| $\ln \ell_\zeta$ | $\mathcal{N}(-0.5, 1)$ | equation 6 |
| $a_\zeta$ | $\mathcal{HN}(4)$ | equation 6 |
| $\beta_q$ | $\mathcal{N}(0, 3)$ | equation 2 |
| $\kappa$ | $\mathcal{N}(0, 6)$ | equation 3 |
| $\chi_{\mathrm{eff,\ max}}$ | $\mathcal{U}(0.05, 1)$ | equation 8 |
| $\chi_{\mathrm{eff,\ min}}$ | $\mathcal{U}(-1, \chi_{\mathrm{eff,\ max}})$ | equation 8 |

**Supplementary Table 1**: Priors adopted for the hyperparameters of the population models.

# 1 Uncertainties about the pair instability mass gap and alternative explanations for the spin transition

We interpret the spin transition and the cliff as connected with the physics of pair-instability supernovae (PISN). In this Section, we discuss how this interpretation might be affected by current uncertainties about stellar evolution and star cluster dynamics.

The theoretical origin of the PISN is the rapid loss of radiation pressure that occurs once the oxygen-rich core of a massive star becomes hot enough for thermal photons to reach energies of order $\sim 1\,\mathrm{MeV}$; at these energies, collisions between energetic photons and atomic nuclei can produce free electron–positron pairs, softening the equation of state. This triggers a thermal runaway in which the core contracts, heats further, and ignites oxygen explosively. Slightly less massive progenitors experience pulsational–PISN instead: repeated contractions and explosive oxygen-burning episodes eject substantial mass but do not fully unbind the star. The cumulative mass loss from these pulsations sets the lower edge of the PISN black-hole mass gap, as the resulting black holes are significantly lighter than their progenitor helium cores.

The lower edge of the pair-instability mass gap is usually assumed to lie at 40–$50\,M_\odot$, based on calculations using pure-helium stellar models—i.e., stars that have lost their hydrogen envelopes from the zero-age main sequence [1, 2]. Although some early studies also examined pair-instability in pure-oxygen stellar models [3], contemporary work typically adopts pure–helium stellar models. The main motivation for this assumption is that massive stars are expected to lose their hydrogen envelopes either through binary interactions or, at sufficiently high metallicity, through strong line-driven winds. Moreover, codes integrating stellar structure encounter less numerical issues if the star does not develop a large hydrogen-rich envelope and/or a sharp core-envelope boundary [2]. However, several authors demonstrated that —even for the fiducial $^{12}\mathrm{C}(\alpha,\gamma)^{16}\mathrm{O}$ rate— the lower edge of the mass gap can be located at a much higher value $70-90\,M_\odot$ in the case of a metal—poor single star that retains a large portion of its H-rich envelope until collapse [1, 2, 4, 5]. Here, low metallicity ($Z < 10^{-3}$) and isolated evolution imply suppressed stellar winds and hence survival of the envelope [6]. Other uncertainties stem from assumptions for convection [7], core overshooting and envelope undershooting that can lead to substantial dredge-up episodes [8], stellar rotation [9], mass loss during pulsational pair instability [10], and onset of shocks during failed supernovae [11].

Scenarios that place the lower edge of the gap significantly above $\sim 60\,M_\odot$ typically require the progenitor to retain at least part of its hydrogen envelope until collapse (but see [12] for a counterexample). This outcome is unlikely in tight binary systems, where envelope stripping during mass transfer naturally produces a sharp drop in the primary-mass distribution above $\sim 40\,M_\odot$ [e.g., Fig. 12 in 13]. Thus, we do not expect isolated binary evolution to populate the region above the cliff. In contrast, very massive metal-poor single stars or stellar-collision products [e.g., 14] can form black holes with masses $\gtrsim 50\,M_\odot$ [e.g., 15], and these channels could also populate the valley near $\simeq 14\,M_\odot$.

Dynamical processes (especially exchanges) in a dense stellar cluster are the most effective mechanisms through which such over-sized single black holes might pair up

with other black holes and merge. This is also compatible with a cliff in the merger rate density at $40\,M_\odot$, as dynamical exchanges involving over-sized black holes are orders of magnitude less common than isolated binary mergers, even in optimistic cases [16].

The most controversial aspect is the black hole spin distribution of such oversized black holes. While there are several reasons to suspect that high spins are possible in this scenario (e.g., limited ejection of mass and angular momentum at low metallicity, spin up of a stellar collision product), a study that investigates the spins of oversized first generation black holes is missing. Considering such uncertainties, we will extend our analysis to first-generation oversized black holes in future works.

Finally, we note that binary mass transfer can produce a cutoff in the secondary-mass distribution at $\sim 40\,M_\odot$ without PISN mass gap [e.g., 17]. Triple interactions could also lead to misaligned systems [e.g., 18, 19], and high spins above the mass gap through consecutive mergers [20], although these latter are thought to be extremely rare. Finally, primordial black holes are another viable interpretation for the population above the cliff. Although traditionally associated with low spins, recent models show that primordial black holes might also achieve large spins [21].

# 2 $\chi_{\rm eff}$ distribution as a Gaussian process

Supplemetary Figure 3 shows the inferred distribution of $\chi_{\rm eff}$ under equation 4 in the Methods section where the $\chi_{\rm eff}$ distribution of the high-mass population is treated non-parametrically using a Gaussian Process prior. For $m_1 < \tilde{m}$, the distribution is well described by a narrow Gaussian with mean $\mu = 0.04^{+0.02}_{-0.02}$ and standard deviation $\log_{10}\sigma = -1.15^{+13}_{-15}$, while for $m_1 > \tilde{m}$ it broadens and becomes consistent with being a uniform distribution that is symmetric around zero. We estimate the posterior probability that the effective spin distribution extends to negative values by evaluating $\mathrm{CDF}(\chi_{\rm eff} = 0)$ for each posterior sample of the non-parametric model. Because the model probability is non-zero across $\chi_{\rm eff}$, we compute the fraction of samples with $\mathrm{CDF}(\chi_{\rm eff} = 0) > 0.01$. This provides a conservative proxy for testing whether the minimum supported $\chi_{\rm eff}$ value is negative. We find that more than 98% of posterior samples satisfy this condition, indicating that the non-parametric inference strongly favors a high-mass binary population that includes systems with negative effective spin.

This is a consistent result recovered among the models presented in this work and shows that a distribution which only contains binaries with aligned spins for the high-mass population is disfavored by current data. We also obtain $\mathrm{CDF}(\chi_{\rm eff} = 0) = 0.29^{+0.30}_{-0.26}$ (90% credibility), indicating that current observations do not place stringent constraints on the symmetry of the $\chi_{\rm eff}$ distribution based on this model.

Finally, we note that excluding the exceptionally massive event GW231123 [22] from our sample, which may involve other channels other than hierarchical mergers or which primary might lie above the PISN mass gap, leads to tighter constraints on the presence of misaligned systems in the high-mass population. In this case, we find that the population contains misaligned systems with negative $\chi_{\rm eff}$ at 99% confidence and that $\mathrm{CDF}(\chi_{\rm eff} = 0) = 0.31^{+0.28}_{-0.27}$.

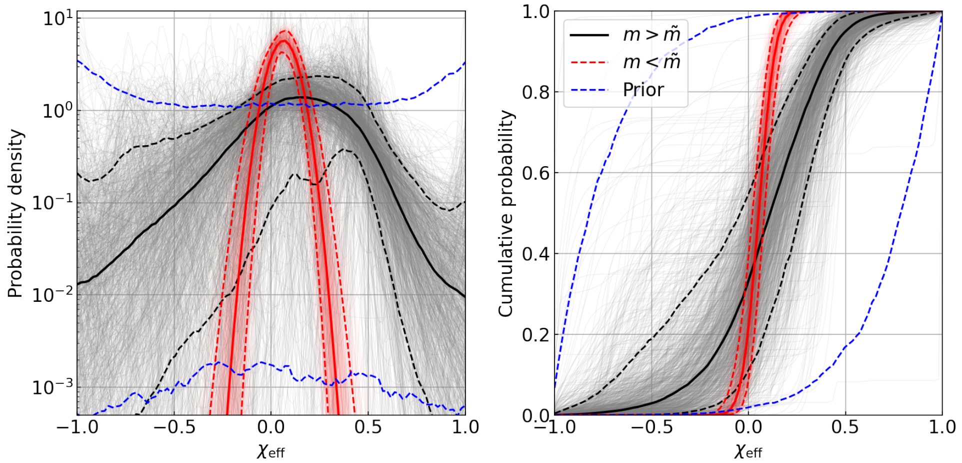


**Supplementary Figure 3**: The distribution of $\chi_{\rm eff}$ for the low and high mass populations under equation 4 in the Methods section. Here the $\chi_{\rm eff}$ distribution of the high-mass population is modeled non-parametrically. Solid lines are median, while dashed lines show 10% and 90% of the distributions. The population below $\tilde{m}$ is represented by a narrow Gaussian inconsistent with hierarchical formation, while the population above $\tilde{m}$ is characterized by a broad $\chi_{\rm eff}$ distribution that is consistent with isotropy (i.e., symmetry around zero) as expected for hierarchical mergers in dynamical environments [23].

# 3 Hierarchical inference

We carry out hierarchical inference on the binary black hole population using Hamiltonian Monte Carlo (HMC) in `numpyro`, a probabilistic programming framework built on `jax`. Within the standard framework of hierarchical Bayesian inference, for each event with posterior $p(\theta_i|d_i)$, the hyperparameter posterior is [e.g., 24–26]

$$p(\Lambda|\{d_i\}) \propto p(\Lambda)\, \xi^{-N_{\rm obs}}(\Lambda) \prod_{i=1}^{N_{\rm obs}} \left\langle \frac{p(\theta_i|\Lambda)}{p_{\rm pe}(\theta_i)} \right\rangle,$$

where $p_{\rm pe}(\theta_i)$ is the prior used in parameter estimation and $\langle\cdot\rangle$ denotes an expectation over posterior samples.

The detection efficiency $\xi(\Lambda)$ is computed using injection campaigns [26–29],

$$\xi(\Lambda) = \frac{1}{N_{\rm inj}} \sum_{i=1}^{N_{\rm found}} \frac{p(\theta_i|\Lambda)}{p_{\rm inj}(\theta_i)}\,,$$

where injections are reweighted from the reference distribution $p_{\rm inj}$ to the proposed model $p(\theta|\Lambda)$.

To mitigate sampling variance, we track the effective number of posterior samples [30],

$$N_{\mathrm{eff},i}(\Lambda) = \frac{\left[\sum_j w_{i,j}(\Lambda)\right]^2}{\sum_j w_{i,j}^2(\Lambda)} \ ,$$

with $w_{i,j}(\Lambda) = p(\theta_{i,j}|\Lambda)/p_{\mathrm{pe}}(\theta_{i,j})$, and the effective number of injections,

$$N_{\mathrm{eff}}^{\mathrm{inj}}(\Lambda) = \frac{\left(\sum_i w_i(\Lambda)\right)^2}{\sum_j w_j^2(\Lambda)} \ .$$

Following [30], we require $N_{\mathrm{eff}}^{\mathrm{inj}} \gtrsim 4N_{\mathrm{obs}}$. We safeguard the inference by penalizing models with $N_{\mathrm{eff}}^{\mathrm{inj}} < 4N_{\mathrm{obs}}$ or $\min \log N_{\mathrm{eff},i} < 0.6$, adding

$$\ln S\left(\frac{N_{\mathrm{eff}}^{\mathrm{inj}}}{4N_{\mathrm{obs}}}\right) + \ln S\left(\frac{\mathcal{N}}{0.6}\right) , \qquad S(x) = \frac{1}{1+x^{-30}} ,$$

to the log-likelihood. This ensures that models with pathologically low effective sample sizes are excluded.

# References


[1] Woosley, S.E.: Pulsational Pair-instability Supernovae. Astrophys. J. **836**(2), 244 (2017)

[2] Farmer, R., Renzo, M., de Mink, S.E., Marchant, P., Justham, S.: Mind the Gap: The Location of the Lower Edge of the Pair-instability Supernova Black Hole Mass Gap. Astrophys. J. **887**(1), 53 (2019)

[3] Fraley, G.S.: Supernovae Explosions Induced by Pair-Production Instability. Astrophys. Space Sci. **2**(1), 96–114 (1968)

[4] Woosley, S.E., Heger, A.: The Pair-instability Mass Gap for Black Holes. Astrophys. J. Lett. **912**(2), 31 (2021)

[5] Hendriks, D.D., van Son, L.A.C., Renzo, M., Izzard, R.G., Farmer, R.: Pulsational pair-instability supernovae in gravitational-wave and electromagnetic transients. Mon. Not. R. Astron. Soc. **526**(3), 4130–4147 (2023)

[6] Vink, J.S., Higgins, E.R., Sander, A.A.C., Sabhahit, G.N.: Maximum black hole mass across cosmic time. Mon. Not. R. Astron. Soc. **504**(1), 146–154 (2021)

[7] Renzo et al.: Massive runaway and walkaway stars. A study of the kinematical imprints of the physical processes governing the evolution and explosion of their binary progenitors. Astron. Astrophys. **624**, 66 (2019)

[8] Costa et al.: Formation of GW190521 from stellar evolution: the impact of the hydrogen-rich envelope, dredge-up, and $^{12}C(\alpha, \gamma)^{16}O$ rate on the pair-instability black hole mass gap. Mon. Not. R. Astron. Soc. **501**(3), 4514–4533 (2021)

[9] Mapelli et al.: Impact of the Rotation and Compactness of Progenitors on the Mass of Black Holes. Astrophys. J. **888**(2), 76 (2020)

[10] Woosley, S.E.: The Evolution of Massive Helium Stars, Including Mass Loss. Astrophys. J. **878**(1), 49 (2019)

[11] Fernández, R., Quataert, E., Kashiyama, K., Coughlin, E.R.: Mass ejection in failed supernovae: variation with stellar progenitor. Mon. Not. R. Astron. Soc. **476**(2), 2366–2383 (2018)

[12] Farag, E., Renzo, M., Farmer, R., Chidester, M.T., Timmes, F.X.: Resolving the Peak of the Black Hole Mass Spectrum. Astrophys. J. **937**(2), 112 (2022)

[13] Iorio et al.: Compact object mergers: exploring uncertainties from stellar and binary evolution with SEVN. Mon. Not. R. Astron. Soc. **524**(1), 426–470 (2023)

[14] Arca Sedda et al.: The DRAGON-II simulations - II. Formation mechanisms, mass, and spin of intermediate-mass black holes in star clusters with up to 1 million stars. Mon. Not. R. Astron. Soc. **526**(1), 429–442 (2023)

[15] Costa et al.: Massive binary black holes from Population II and III stars. Mon. Not. R. Astron. Soc. **525**(2), 2891–2906 (2023)

[16] Santoliquido et al.: The Cosmic Merger Rate Density Evolution of Compact Binaries Formed in Young Star Clusters and in Isolated Binaries. Astrophys. J. **898**(2), 152 (2020)

[17] Belczynski et al.: Evolutionary roads leading to low effective spins, high black hole masses, and O1/O2 rates for LIGO/Virgo binary black holes. Astron. Astrophys. **636**, 104 (2020)

[18] Antonini, F., Rodriguez, C.L., Petrovich, C., Fischer, C.L.: Precessional dynamics of black hole triples: binary mergers with near-zero effective spin. Mon. Not. R. Astron. Soc. Lett. **480**(1), 58–62 (2018)

[19] Stegmann, J., Klencki, J.: Spin-orbit misalignment and residual eccentricity are evidence that neutron star-black hole mergers form through triple star evolution. arXiv e-prints, 2506–09121 (2025)

[20] Liu, B., Lai, D.: Hierarchical black hole mergers in multiple systems: constrain the formation of GW190412-, GW190814-, and GW190521-like events. Mon. Not. R. Astron. Soc. **502**(2), 2049–2064 (2021)

[21] De Luca, V., Franciolini, G., Riotto, A.: GW231123: a Possible Primordial Black

Hole Origin. arXiv e-prints, 2508–09965 (2025)

[22] Abac, e.a. A. G.: GW231123: a Binary Black Hole Merger with Total Mass 190-265 $M_{\odot}$. arXiv e-prints, 2507–08219 (2025)

[23] Rodriguez, C.L., Zevin, M., Pankow, C., Kalogera, V., Rasio, F.A.: Illuminating Black Hole Binary Formation Channels with Spins in Advanced LIGO. Astrophys. J. Lett. **832**(1), L2 (2016)

[24] Fishbach, M., Holz, D.E., Farr, W.M.: Does the black hole merger rate evolve with redshift? Astrophys. J. Lett. **863**(2), 41 (2018)

[25] Mandel, I., Farr, W.M., Gair, J.R.: Extracting distribution parameters from multiple uncertain observations with selection biases. Mon. Not. R. Astron. Soc. **486**(1), 1086–1093 (2019)

[26] Callister, T.A., Miller, S.J., Chatziioannou, K., Farr, W.M.: No Evidence that the Majority of Black Holes in Binaries Have Zero Spin. Astrophys. J. Lett. **937**(1), 13 (2022)

[27] Abbott et al.: GWTC-2.1: Deep extended catalog of compact binary coalescences observed by LIGO and Virgo during the first half of the third observing run. Phys. Rev. D **109**(2), 022001 (2024)

[28] The LIGO Scientific Collaboration, Virgo Collaboration, and KAGRA Collaboration: GWTC-3: Compact Binary Coalescences Observed by LIGO and Virgo During the Second Part of the Third Observing Run — O1+O2+O3 Search Sensitivity Estimates. Zenodo, 10.5281/zenodo.7890398 (2023).

[29] Essick et al.: Compact binary coalescence sensitivity estimates with injection campaigns during the LIGO-Virgo-KAGRA Collaborations' fourth observing run. Phys. Rev. D **112**(10), 102001 (2025)

[30] Essick, R., Farr, W.: Precision Requirements for Monte Carlo Sums within Hierarchical Bayesian Inference. arXiv e-prints, 2204–00461 (2022)
