# Supplementary material for: Gravitational-wave constraints on the pair-instability mass gap and nuclear burning in massive stars
Source: Nat Astron. 2026 May 7;10(7):1049–56. doi: 10.1038/s41550-026-02847-0 (PMC13379312; doi:10.1038/s41550-026-02847-0)
Supplement: Supplementary file 2 — Peer Review File [file 41550_2026_2847_MOESM2_ESM.pdf]

nature portfolio

Peer Review File

Gravitational-wave constraints on the pair-instability mass gap and nuclear burning in massive stars

Corresponding Author: Professor Fabio Antonini

This manuscript has been previously reviewed at another journal. This document only contains information relating to versions considered at Nature Astronomy.

This file contains all reviewer reports in order by version, followed by all author rebuttals in order by version.

Version 0:

Reviewer comments:

Reviewer #1

(Comments for the Author)
The authors have addressed my previous concerns. While alternative formation scenarios remain plausible and, in my view, the current data are not yet sufficient to fully confirm a PISN-based interpretation, the manuscript presents an interesting and physically well-motivated analysis of the gravitational-wave population.
Overall, the proposed scenario offers a compelling explanation for trends observed in, however, the still-limited sample of detections, effectively synthesizing insights gained over roughly a decade of gravitational-wave observations. The results are timely, relevant, and likely to have broad impact. Subsequent related works generally show consistent findings. The study has already attracted significant attention. I therefore consider the manuscript suitable for publication in Nature Astronomy, pending minor revisions.

Minor points to address:
1. Please report in the manuscript the median ⟨χ_eff⟩ values for all models and for both high and low-mass population, not only the model that favors more the proposed scenario, to provide a complete and transparent picture.
2. Include a brief discussion of Ray & Kalogera (2025, arXiv:2510.18867), noting potential tensions or consistencies with the presented results.
3. Correct the reference for the isolated binary evolution scenario (with high PSN limit) to Olejak et al., 2022 (arXiv:2204.09061), particularly see Figs. 5 and 7 with m1 and m2 distributions.
4. Please soften the wording in the Abstract (and, where relevant, in the main text) to better reflect the current level of statistical evidence. As the authors themselves note, the χ_eff distribution of the high-mass population appears consistent with being symmetric about zero, with no statistically significant preference for aligned systems. This remains suggestive rather than conclusive, and the language should reflect that the evidence is not yet very strong.
5. Note missing keywords in the abstract.

Recommendation: Accept after minor revisions. I would appreciate seeing the revised manuscript before final acceptance.

Reviewer #2

(Comments for the Author)
I thank the authors for addressing my previous comments. On the point of novelty, I appreciate the detailed reply and justification of the salient new features of the current manuscript. The authors write "the significance increased [relative to the GWTC-3 result] because the new data reduce the posterior probability of models without a transition." However, their analysis does not show this conclusively, because they do not consider models without a transition. This likely relates to my previous question about the quoted Bayes factor, which I do not think has been sufficiently addressed. In general, the manuscript would benefit from a more detailed comparison of the GWTC-4 vs GWTC-3 results in the text, which could draw on the argument presented in point 3 of the authors' reply to my original comments. The discussion starting on L122 is qualitative and does not discuss specifically how/why the GWTC-4 results present an improvement over the GWTC-3 constraints.

As another general comment, the paper would benefit from a discussion of other concurrent works that make statements about the presence/absence of the PISN mass gap and its implications for the astrophysical S-factor (Ray+ https://arxiv.org/pdf/2510.18867, Tong+ https://arxiv.org/pdf/2509.04151) to place this work in the larger current context of the field.

If the authors are willing to add these additional discussions to help contextualize the novelty of the work in the manuscript itself and address my detailed comments below, I believe the manuscript is suitable for publication in Nature Astronomy.

Detailed comments
- The calculation of the Bayes factor in favor of the transition mass model has not been sufficiently explained. No such model without the transition mass is described in the methods. Are the authors repeating the inference with the LVK default model to calculate this Bayes factor? If so, this should be described somewhere in the text. Otherwise, are the authors using the model evidence directly reported by the LVK for this comparison? This does not enable an apples-to-apples comparison, unless they are using the same software with the same initial seed set by the LVK.

- In the discussion of the result of the parametric chi_eff model around L115, the authors say that only hierarchical mergers predict a cutoff at |chi_eff| < 0.5. I understand that this is derived in Ref 42, but the reader would benefit from a short (one-two sentence) qualitative explanation of how hierarchical mergers predict this cutoff astrophysically.

- It is encouraging to see that the GP chi_eff model is flexible enough to recover a narrow distribution, if that were preferred by the data. I recommend that the authors mention this directly in the text above Eq 4 when the model is introduced for the sake of clarity. I also wonder if the results of this model could be used to directly address the question of whether the transition is required, by calculating some quantifiable measure of the difference between the narrow, low-spin Gaussian and the broad GP component at high masses (JD divergence, Hellinger distance, characteristic width, etc.).

- In L441, the authors write "We infer that chi_eff,min >0 at 98% confidence under the non-parametric model, providing evidence for the presence of misaligned spins (chi_eff <0) in the high-mass binary population." I do not understand this reasoning. If the minimum value of chi_eff in the population is positive at 98% credibility, then doesn't this strongly disfavor the presence of a significant misaligned spin population? Is this a typo, and it should say "chi_eff,min *<* 0 at 98%"? Also, the authors should add an explanation of what chi_eff,min means for the nonparametric model from Eq 4 shown in Fig A1. This parameter is only introduced in the context of the parametric model, so there is no unambiguous definition of chi_eff,min for the nonparametric model.

- From the new Appendix B, am I to understand that the model in Eq 4 is never actually used in any analysis? If that's the case, this equation should be replaced with Eqs B1-B2 directly in the main text. Even with this new text and the additional sentence in the caption of Fig. 1, it is still not clear how the two mass populations in Fig 1 are constructed. I recommend adding an equation describing the weighting process.

Typos
- L185: "where the 90% bound of the mixing *fraction* rises"
- There is no predicate in the sentence beginning on L295 with "If there is a mass m_* …"
- L335: "For each posterior tracer" -> "For each posterior trace"

Open Access This Peer Review File is licensed under a Creative Commons Attribution 4.0 International License, which permits use, sharing, adaptation, distribution and reproduction in any medium or format, as long as you give appropriate credit to the original author(s) and the source, provide a link to the Creative Commons license, and indicate if changes were made.
In cases where reviewers are anonymous, credit should be given to 'Anonymous Referee' and the source.
The images or other third party material in this Peer Review File are included in the article's Creative Commons license, unless indicated otherwise in a credit line to the material. If material is not included in the article's Creative Commons license and your intended use is not permitted by statutory regulation or exceeds the permitted use, you will need to obtain permission directly from the copyright holder.
To view a copy of this license, visit https://creativecommons.org/licenses/by/4.0/

REPLY to R1:

1. We have now reported the median/mean Xeff for both models in the manuscript, and for both high and low mass populations; for the low mass population we reported the mean of the distributions as this is a more natural parameter for the Normal model adopted. We also reported the sigma’s of the Xeff distributions below the transition. See paragraphs starting in line 77, and 112.

2. We have added a comment to Ray & Kalogera in line 231 and after.

3. The reference has been added in the first paragraph of the article.

4. In the abstract, we replaced `strong evidence'' with `evidence'' and softened the wording accordingly. Generally, we moderated the conclusions throughout the manuscript, while preserving the central point that the data are consistent with the dynamical hypothesis, rather than claiming that they demonstrate it.

5. keynotes have been removed as for journal requirement.

REPLY to R2

We have added a more quantitative comparison to the GWTC-3 results, as suggested. We focus this discussion on the better constrained shape of the Xeff distribution, as suggested. See paragraph starting in line 130. We now mention the mass-gap result of the concurrent work by Tong+2025 and added a citation to this work. We have added a brief discussion of Ray+ results in line 231 and after.

Detailed R2 comments reply:

A- We have now clarified that the Bayes factor is calculated relative to the standard LVK model. We have repeated the inference using the same machine, software and set up as for the other models with a mass transition. See line 91.

B- We have now clarified why second generation mergers leads to Xeff<0.5. See text around line 64.

C- We have now clarified below Eq.~4 that the GP model is flexible enough to recover a narrow distribution if preferred by the data. We thank the referee for suggesting a quantitative assessment of the separation between the low-mass narrow component and the high-mass GPinferred distribution as a function of mass. Although we do not include this analysis here, we agree it is a useful direction for future work and one that deserves a dedicated study, as a careful definition and interpretation of such metrics would go beyond the scope of the present paper.

D- In former Line 441 there was indeed a typo which we now have corrected. We have opted for not using chi_eff,min when describing the results of the nonparametric model in Supplementary Information, and indeed refrase the all discussion to avoid confusion with the chi_eff,min parameter used in the uniform model.

E- We have replaced the equations as suggested. We have now clarified how the two mass distributions are constructed in the caption of Fig. 1.

Typos have been fixed.

Thank you!
